# A role for core planar polarity proteins in cell contact-mediated orientation of planar cell division across the mammalian embryonic skin

**DOI:** 10.1038/s41598-017-01971-2

**Published:** 2017-05-12

**Authors:** Fazal Oozeer, Laura L. Yates, Charlotte Dean, Caroline J. Formstone

**Affiliations:** 1 MRC Centre for Developmental Neurobiology, New Hunts House, Kings College London, London, SE1 1UL UK; 2 MRC Harwell, Oxfordshire, OX11 0RD UK; 3 Department of Biological and Environmental Sciences, University of Hertfordshire, College lane, Hatfield, AL10 9AB UK; 4 Department of Medicine, Imperial College London, London, SW7 2AZ UK

## Abstract

The question of how cell division orientation is determined is fundamentally important for understanding tissue and organ shape in both healthy or disease conditions. Here we provide evidence for cell contact-dependent orientation of planar cell division in the mammalian embryonic skin. We propose a model where the core planar polarity proteins Celsr1 and Frizzled-6 (Fz6) communicate the long axis orientation of interphase basal cells to neighbouring basal mitoses so that they align their horizontal division plane along the same axis. The underlying mechanism requires a direct, cell surface, planar polarised cue, which we posit depends upon variant post-translational forms of Celsr1 protein coupled to Fz6. Our hypothesis has parallels with contact-mediated division orientation in early *C*. *elegans* embryos suggesting functional conservation between the adhesion-GPCRs Celsr1 and Latrophilin-1. We propose that linking planar cell division plane with interphase neighbour long axis geometry reinforces axial bias in skin spreading around the mouse embryo body.

## Introduction

Horizontal (planar) cell divisions that generate symmetric daughters contribute new cells to epithelia thus driving tissue shape and growth. How planar cell division orientation is regulated is of significant interest therefore particularly because defects in this process may contribute to organ malformation and tumourigenesis. Intrinsic and extrinsic cell polarity cues are well-studied and known to play an important role^1^. The complex interplay between planar cell division orientation and interphase cell shape however is not well understood. ‘Hertwig’s’ rule^2^ states that ‘a cell divides along its longest axis’ setting out one mechanism by which cell shape during interphase can determine the orientation of the cleavage plane during a subsequent cell division. The cell microenvironment also exerts a significant influence. Compelling evidence suggests that as mitotic cells round up they retain a ‘memory’ of the spatial geometry of their previous interphase existence by using cellular landmarks derived from both extracellular matrix contacts^3^ and tri-cellular junctions^4^. Another intriguing idea posits that local cell shape during interphase influences the cleavage plane of neighbouring mitotic cells^5^. In this study we provide evidence for a cell contact-dependent mechanism of cell division orientation in the mammalian skin epithelium, where planar polarity proteins align the cleavage plane of horizontal cell divisions with the planar cell shape of neighbouring interphase cells. We propose a model whereby cell surface asymmetry of planar polarity proteins communicates interphase long axis geometry to a neighbouring dividing cell to directly orient the mitotic spindle.

## Results

### Orientation of skin planar oriented cell division is dependent upon Celsr1 and fz6

**Figure 1:**
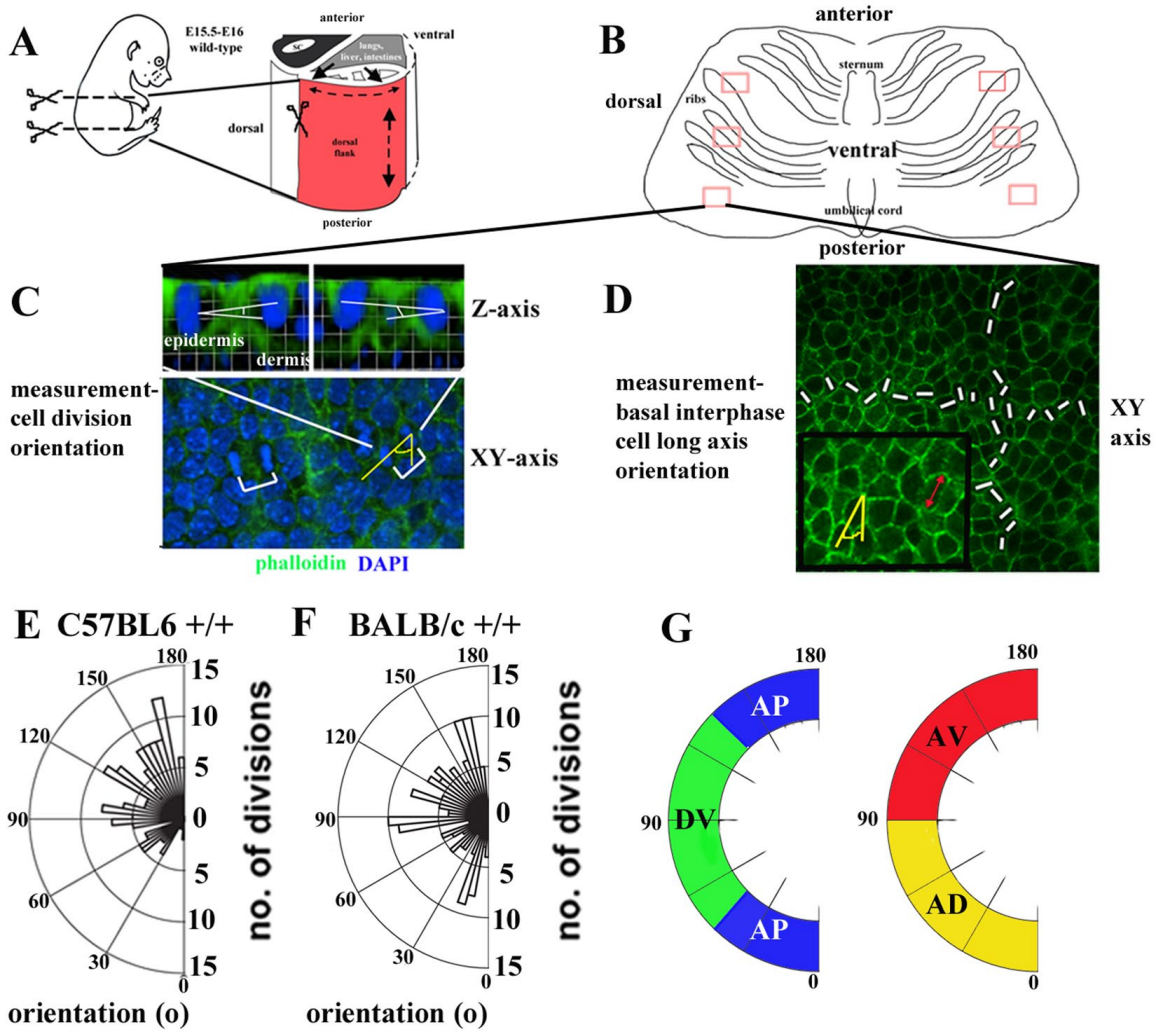
A role for core proteins in planar cell division orientation across the mammalian epidermis. (**A**) black arrows highlight the circumferential growth of the embryo, dashed arrows highlight assumed axial skin spreading. Scissors indicate dissection area. (**B**) 10 images were taken within each anterior, mid and posterior region of the dorsal flank. (**C**,**D**) Top panels in (**C**) are 3D reconstructions of individual mitoses. Lower panel in (**C**) brackets label cell divisions in telophase and cytokinesis. White lines in (**D**) show method of sampling interphase long axis for each image. Inset shows method of measuring interphase long axis orientation with respect to the anterior-posterior axis (yellow) as well as the extent of the long axis (double-headed red arrow). (**E**,**F**) polar plots of total planar oriented cell divisions across the skin epithelium, E16 wild-type embryos from two different mouse strains are shown. Concentric circles on polar plots label multiples of n = 5 divisions. Radial spokes denote angle of division orientation. (**G**) schematic of quadrants used for statistical analysis. Blue quadrant denotes anterior-posterior (AP; 0–45° and 135–180°). Green quadrant denotes dorso-ventral (DV: 45°–135°). Red quadrant denotes anterior-ventral (AV; 90–180°) and yellow quadrant denotes anterior-dorsal (AD; 0–90).

**Figure 2:**
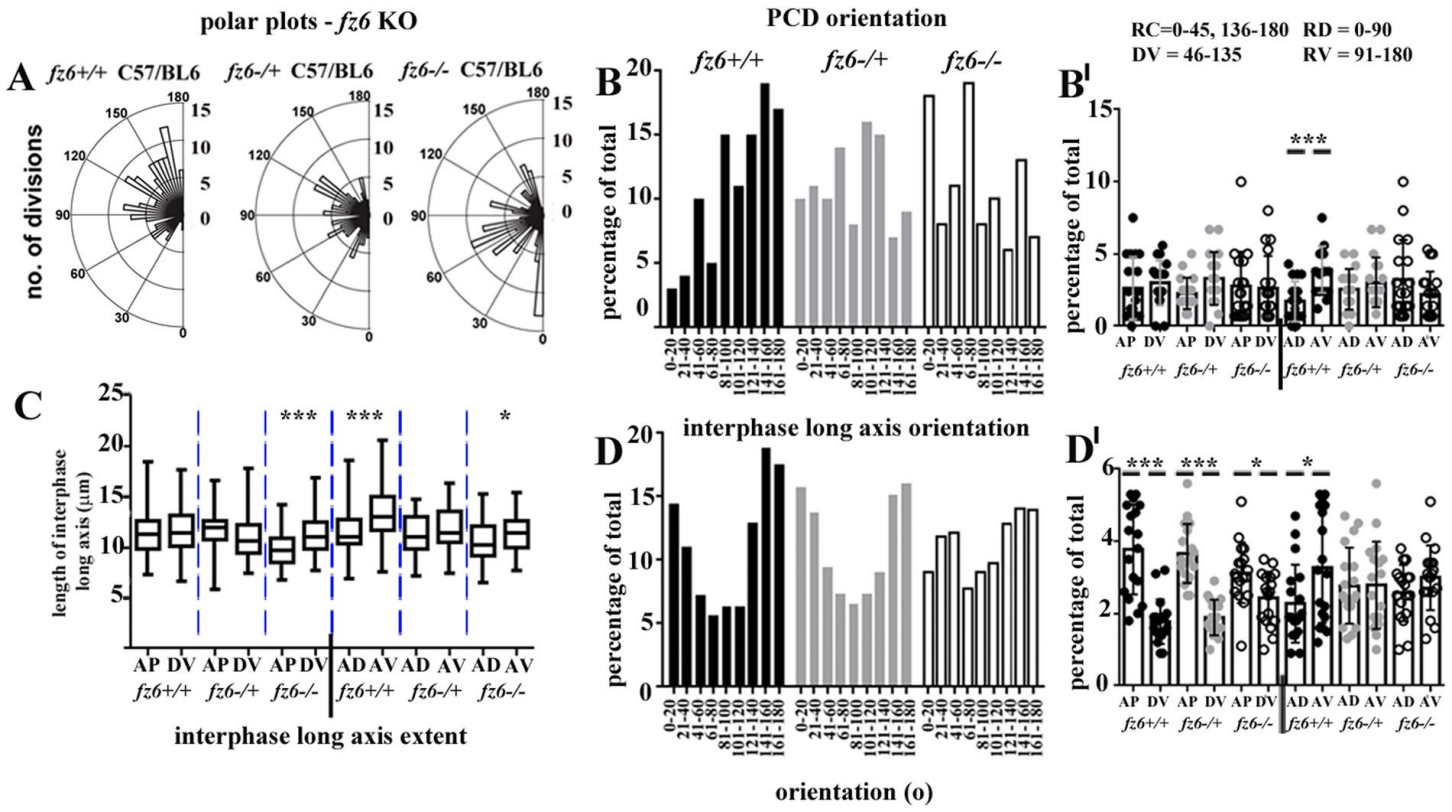
A role for Fz6 in planar cell division orientation across the mammalian epidermis. (**A**) polar plots of total planar oriented cell divisions from E16 *fz6* knockout litters. The pattern at E15.5 is shown in Supplementary Fig. 1A–C. Blind analyses were performed for all *fz6 KO* littermates, >4 embryos from 3 litters for each group. (**B**,**D**) histograms of PCD and long axis orientation, range of bin widths is shown along the X-axis. (**B**,**D’**) scatter plots of PCD and long axis orientation, mean and SD are shown. (**C**) whisker box plots showing the extent of the longest axis (length between the two shortest opposing interfaces) of basal epithelial cells for each condition. Box plots show maximum and minimum, median and 75% and 25% percentile values. Statistical analysis was Student’s t-test, *denotes P-value > 0.01; ***denotes P-value > 0.0001.

**Figure 3:**
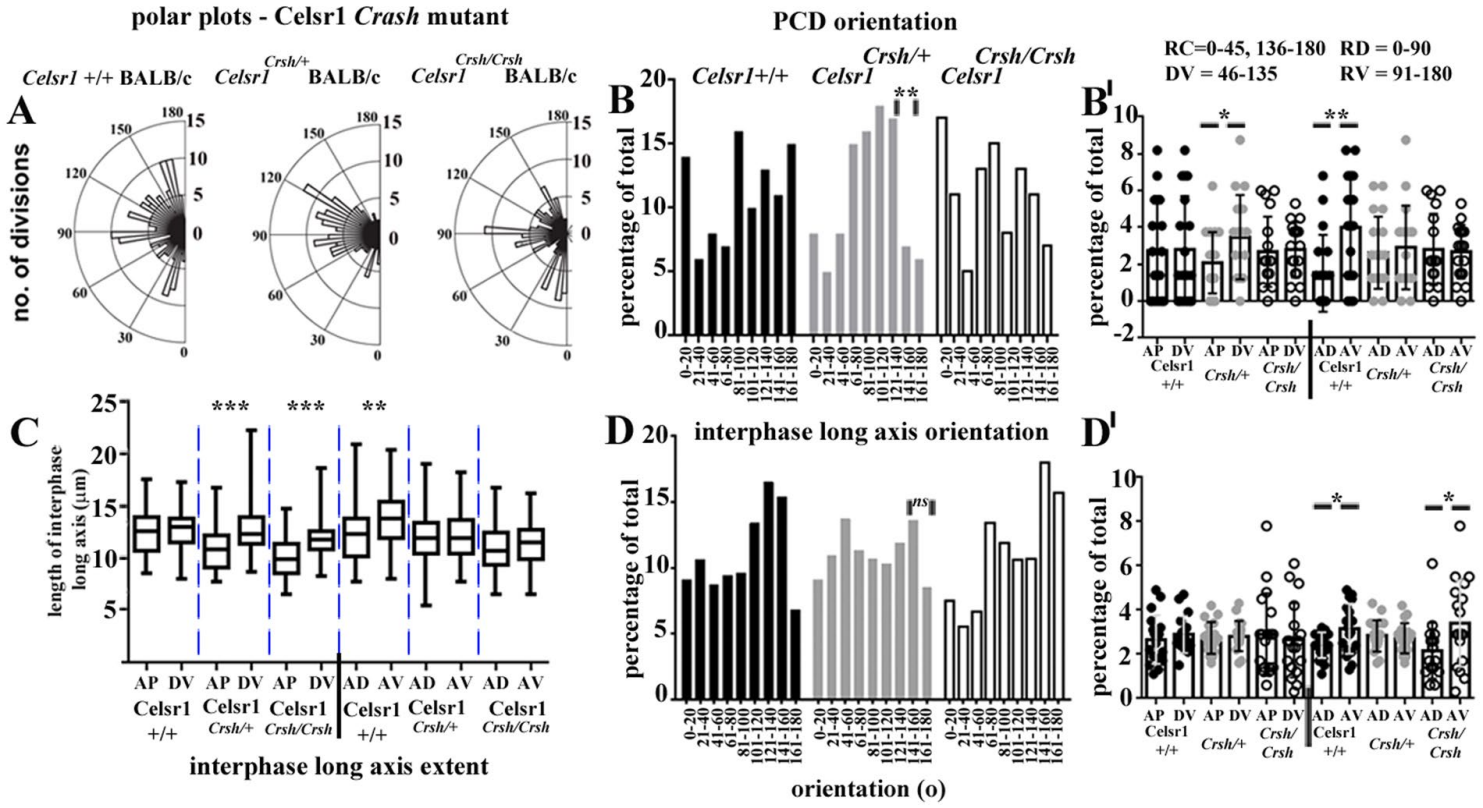
A role for Celsr1 in planar cell division orientation across the mammalian epidermis. (**A**) polar plots of total planar oriented cell divisions in E16 *Celsr1 Crsh* mutant litters. E15.5 litters exhibited a similar pattern to E16 (data not shown). Blind analyses were performed for wild-type and heterozygote *Crsh* littermates, >4 embryos from 4 litters for each group and for each wild-type. (**B**,**D**) histograms of PCD and long axis orientation, range of bin widths is shown along the X-axis. (**B**,**D’**) scatter plots of PCD and long axis orientation, mean and SD are shown. (**C**) whisker box plots showing the extent of the longest axis (length between the two shortest opposing interfaces) of basal epithelial cells for each condition. Box plots show maximum and minimum, median and 75% and 25% percentile values. Statistical analysis was Student’s t-test, *denotes P-value > 0.01; **denotes P-value > 0.001; ***denotes P-value > 0.0001.

Horizontal (planar) cell divisions (PCDs) contribute new cells to the progenitor epithelium of the developing skin^6^. Notably, the molecular pathways of planar polarity have roles in PCD orientation^7–12^ and planar polarity, via the activity of a core planar polarity pathway, is evident in the skin during embryonic development^13^. We addressed therefore whether mammalian orthologues of core planar polarity components (hereafter core proteins), Flamingo:Stan and Frizzled^14–17^, play a role in skin PCD orientation. To this end, we analysed the *Celsr1* mouse mutant *Crash- Crsh* ^18^; and the *fz6* knockout- *fz6* KO^19^; respectively. Notably in the former mutant, which carries a missense mutation in the extracellular domain, Celsr1^*Crsh*^ protein is expressed but not distributed correctly^13, 20^ and is most probably dominant-acting. We examined E15.5-E16 skins when planar polarity is apparent in the organised down-growth of developing HFs^13^ but the skin surface remains relatively flat. We focussed on dorsal flank skin (Fig. 1A; area of skin examined is highlighted in red) and not back skin as in previous studies of epidermal planar polarity^13, 21^ which enabled us to analyse *Crsh* mutant embryos which exhibit an open neural tube and have no back skin covering^18^. Skin was dissected in one piece away from the embryo body (Fig. 1A), immunostained in wholemount and flatmounted for imaging analysis (Fig. 1B). We took ten consecutive confocal images across different rostral-caudal regions of dissected dorsal flank skin for each condition: representative areas are highlighted as red boxes in Fig. 1B. Horizontal divisions were identified in XY slices (defined as oriented <30° to the basal lamina; 6) by generating a Z-stack of each telophase division using Volocity software and measuring the angle of division orientation with respect to the basal lamina, which was discerned using E-cadherin staining which labels the epidermis but not the dermis (Fig. 1C). The angle between the plane of chromatin segregation and the anterior-posterior (AP) axis of the dorsal flank was then determined in the XY plane (Fig. 1C). We expressed our findings as polar plots showing the number of cell divisions (*y*-*axis*) oriented at particular angles (*x*-*axis*; 0–180° expressed as 5° bins) away from the AP axis (Fig. 1E,F). Our findings in wild-type revealed strain-specific differences in PCD orientation at E16 (Fig. 1E,F). Specifically in C57BL6 skins, PCD was enriched in a quadrant on our polar plots, which we designated as anterior ventral (Fig. 1E,G, red quadrant, AV, 90–180°). We designated the orthoganol quadrant anterior dorsal (Fig. 1G, yellow quadrant, AD, 0–90°). A plot of the total number of divisions assigned within 20° bin widths confirmed a bias towards the AV quadrant in wild-type C57BL6 skins (Fig. 2A,B) but also exposed a similar trend towards AV in BALB/c wild-types (Fig. 3A,B). After plotting the proportion of total divisions in 5° bin intervals for the different quadrants and comparing AP (Fig. 1G, blue quadrant, 0–45° and 135–180°) with DV (Fig. 1G, green quadrant, 45–135°) and AD (0–90°) with AV (90–180°) we observed a statistically significant bias in AV versus AD orientation in both wild-type strains (Figs 2A,B’ and 3A,B’). The lack of significant bias AP versus DV across dorsal flank skin from either mutant (Figs 2A,B’ and 3A,B’) was consistent with a previous report^21^.

Surprisingly in *Crsh* heterozygotes, DV orientations predominated at the expense of AP orientations whereas the representation of AD versus AV quadrant PCDs was more similar (Fig. 3A,B’). We observed no axial bias in *fz6* KO heterozygotes (Fig. 2A,B’). In contrast, both *Crsh* and *fz6* KO homozygotes exhibited an increase in AP divisions and a trend towards AD orientations (Figs 2A,B’ and 3A,B’). PCD orientation also exhibited bias towards the AD quadrant in *fz6* KO homozygote skins at E15.5 (Supplementary Fig. S1A–C). Taken together these data are consistent with a role for Celsr1 and Fz6 in orienting PCD across the mammalian embryonic skin.

### Skin PCD orientation is linked to the long axis geometry of neighbouring interphase cells

The geometry of the cell long axis during interphase can act as a default mechanism to direct the plane of a subsequent cell division^2–4^. Core proteins have been shown to influence epithelial planar cell shape^22^, it was possible therefore that Fz6 and Celsr1 might promote alignment of PCDs along particular body axes via general effect(s) on interphase long axis topology. To test this hypothesis we measured both the orientation of progenitor cell long axes orientation and their extent (the distance along the long axis i.e. between the shortest interfaces, labelled by a red-double headed arrow in Fig. 1D). Interphase progenitor cells were sampled from the same images we had used for analysis of PCD orientation (methodology shown in Fig. 1D).

The differential patterns of long axis orientation in C57BL6 wild-types compared to BALB/c were consistent with the strain-specific differences observed for PCD orientation (compare Fig. 2D,D’ with Fig. 3D,D’). In C57BL6 wild-types AP-long axis orientations predominated over DV. Overall however, orientations in the AV quadrant were favoured over AD (Fig. 2D,D’) and long axis extent was similarly biased (Fig. 2C). These data are consistent therefore with the AV bias in PCD orientation (Fig. 2A,B’). BALB/c wild-types also exhibited an AV bias in both long axis orientation (Fig. 3D,D’) and long axis extent (Fig. 3C) consistent with their AV bias in PCD orientation (Fig. 3B,B’).

*Fz6* heterozygotes however exhibited no significant bias in long axis extent (Fig. 2C) or long axis orientation (Fig. 2D,D’), which was consistent with their PCD phenotype (Fig. 2A,B’). Strikingly, DV oriented long axes were the most extended in *Crsh* heterozygotes (Fig. 3C) although any bias in long axis orientation was lost (Fig. 3D,D’). The DV bias in long axis extent however mirrored the DV bias in PCD orientation in this mutant (Fig. 3A,B’). Conversely, we found little correlation between long axis geometry and PCD orientation in homozygous mutants from both strains (Figs 2 and 3). The bias in long axis extent was most significant along the DV axis in *fz6 KO* homozygotes (Fig. 2C) whereas long axis orientation favoured AP alone as observed in heterozygote littermates (Fig. 2D,D’). PCD orientation in *fz6 KO* homozygotes however showed a trend towards AD (Fig. 2A,B’). In *Crsh* homozygotes, the bias in long axis extent was also most significant along the DV axis (Fig. 3C) but long axis orientation favoured AV as it did in wild-type littermates (Fig. 3D,D’). PCD orientation in *Crsh* homozygotes however was unbiased (Fig. 3B,B’). Altogether these data raise the intriguing hypothesis that in wild-type and heterozygote core protein mutants, orientation of skin progenitor cell division correlates with the long axis geometry of neighbouring interphase cells. The data further suggest that this correlation is lost in the homozygote condition.

### Evidence of diversity in core planar polarity protein signalling in mammalian skin

**Figure 4:**
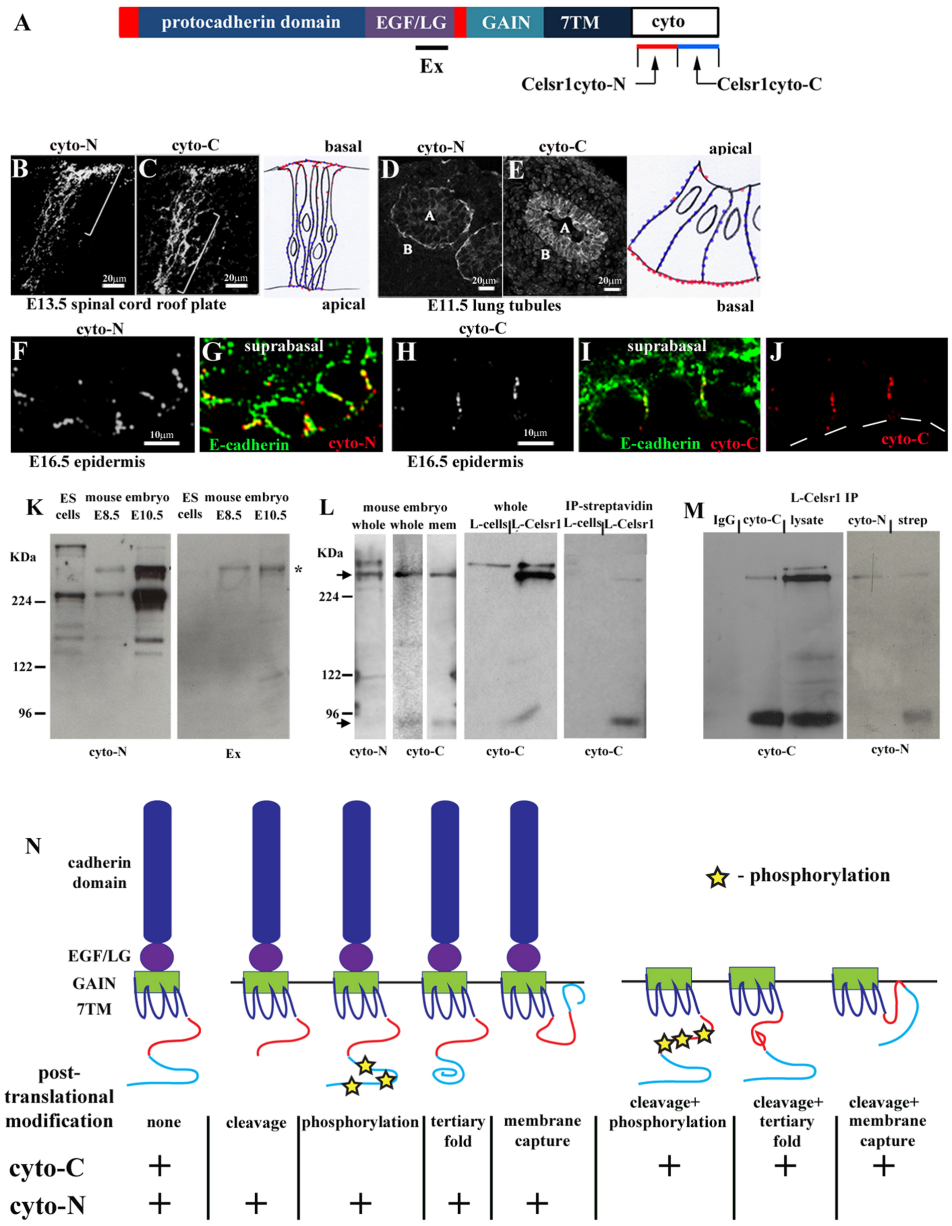
Cellular and molecular evidence for post-translational variants of Celsr1 protein. (**A**) Schematic of Celsr1 protein. Cysteine-rich domains (red), EGF; epidermal growth factor-like domain, LG; laminin-G like domain, GAIN; GPCR auto-proteolytic inducing domain, 7TM; seven-pass transmembrane domain, cyto tail; cytoplasmic tail. Regions of the cytoplasmic tail against which Celsr1-cyto-N and Celsr1-cyto-C antibodies were raised are marked by red and blue bars respectively. A polyclonal antibody against an extracellular LG domain is also shown. (**B**–**I**) immunostaining of frozen sections from different mouse tissues, (**B**–**E**) transverse sections (**F**–**I**) longitudinal sections. (**B**,**C**) representative images, n > 3. Consecutive frozen sections of the developing spinal cord were used to compare cyto-N and cyto-C staining patterns. Brackets label zones of cyto-N and cyto-C enrichment within the roof plate of the embryonic spinal cord. Boxed area outlines roof plate region, dorsal is to the top. Schematic to right of (**B**,**C**) highlights zones of cyto-N (red) and cyto-C (blue) enrichment in roof-plate neuroepithelial cells. (**D**,**E**) representative images, n > 3. A labels apical domain of tubule, B labels basal domain of tubule. Schematic to right of (**D**,**E**) highlights zones of cyto-N (red) and cyto-C (blue) enrichment in lung tubule cells. (**F–J**) representative images, n > 3. White lines denote basal lamina underlying Celsr1-expressing basal progenitor cells (basal monolayer). (**K**) the same tissue extracts were used for Western blot analysis of cyto-N and Ex antibodies. Asterisk labels Celsr1 p400 protein, n = 3. (**L**) L-Celsr1 denotes stable Celsr1 expressing L cells, IP is immunoprecipitation, n = 3 for each set of blots. Arrows mark Celsr1 p400 and p85 protein species. (**M**) Negative control was IgG. Specific binding of Celsr1 antibodies to Western blots was visualised using Protein A-HRP, n = 3 for each set of blots. Notably, cyto-N recognised denatured p85 when cell surface-tagged proteins were enriched from the same batch of Celsr1-expressing cells as immunoprecipitation reactions. (**N**) schematic showing predicted post-translational modifications of Celsr1 protein which we believe may underpin the differential recognition of native Celsr1 protein by cyto-C and cyto-N antibodies. Both antibodies could recognise a non-modified protein. Post-translational modifications could remove or block antibody-specific epitopes of the native Celsr1 protein. Thus cyto-C may not favour some large Celsr1 protein species because of C-terminal cleavage, site-specific phosphorylation, a change in tertiary protein folding or membrane capture of the C-terminal cytoplasmic tail. Equally, similar mechanisms linked to the N-terminal cytoplasmic tail of Celsr1 could prevent cyto-N-binding to p85 in its native form following protein cleavage. Membrane capture of the cytoplasmic tail of 7TM proteins can occur via post-translational modification of C-terminal tail cysteines with palmitic acid. Such palmitoylation could create a new intracellular loop^48^ to regulate 7TM protein endocytosis and trafficking^49^. Annotated Celsr1 tail sequence is shown in Supplementary Fig. S2H.

Communication of interphase long axis geometry to a neighbouring mitotic cell would require a direct cell surface cue. In particular tissue contexts Fz proteins have been shown to act at the cell surface to orient spindle orientation^11, 23, 24^. In skin however, Fz6, along with Celsr1, disappears from the cell surface of basal progenitor cells during mitosis and accumulates within intracellular puncta only to be redelivered to the cell surface at cytokinesis^21^. In seeking to understand how core proteins could connect cell division plane to neighbouring interphase cell shape we revisited our findings from a previous study. A Celsr1-specific polyclonal antibody raised against the N-terminal region of the Celsr1 cytoplasmic tail, in both lung and neural tissue had raised the novel hypothesis that variant Celsr1 protein species co-exist within mammalian epithelia^20, 25, 26^. To further explore these observations we generated two polyclonal antibodies against the Celsr1 cytoplasmic tail which recognised either N-terminal tail (cyto-N antibody) or C-terminal tail (cyto-C antibody) epitopes (Supplementary Fig. S2). Notably, cyto-N was raised against the same Celsr1 tail region as the previous antibody^20^ and was indistinguishable from it in all assays performed both *in vivo* and *in vitro*. *In vitro* data are shown in Supplementary Fig. S2 and demonstrate that both cyto-N and cyto-C antibodies recognise the same Celsr1 gene product by immunohistochemistry. *In*-*vivo*, cyto-N staining of the embryonic spinal cord (Fig. 4B; Supplementary Fig. S2) and lung (Fig. 4D) mirrored our previous findings^20, 26^. In embryonic skin, however, cyto-N labelled Celsr1 proteins distributed predominantly along lateral cell membranes of the basal epithelium (Fig. 4F,G). Conversely, cyto-C consistently labelled lateral cell membranes in all embryonic epithelia analysed (Fig. 4C,E,H,I,J). Thus in skin, unlike other epithelia, the patterns of cyto-C and cyto-N staining are highly similar. Importantly, they both label Celsr1 proteins located within a sub-cellular compartment associated with skin core planar polarity signalling^13^.

The differential staining patterns of cyto-C and cyto-N were highly intriguing and suggested recognition of distinct Celsr1 proteins. We had previously reported two Celsr1 protein species of molecular weight, namely p400 and p85^20^. As with our other N-terminal Celsr1 tail antibody, cyto-N recognised the same p400 product in denatured protein preparations from whole mouse embryo tissue (asterisk, Fig. 4K, arrow Fig. 4L) as did a Celsr1-specific polyclonal antibody raised against the Celsr1 extracellular domain (Fig. 4A,K; refs 20 and 25). Intriguingly, in the same whole embryo extracts, cyto-C additionally recognised a membrane-enriched species, p85 (arrows, Fig. 4L), which is most probably a cleaved form of Celsr1. Cyto-C also recognised both p400 and p85 in extracts from whole Celsr1-expressing L cells (Fig. 4L). Conversely, enrichment for cell surface proteins, either by sucrose gradient separation of sub-cellular fractions or using a cell surface biotinylation strategy was necessary to enable both our N-terminal tail antibodies (Celsr1cyto and cyto-N) to visualise p85 in denaturing SDS-PAGE gels (right hand panel, Fig. 4M and ref. 20). Immunoprecipitation from whole cultured cell lysates revealed that cyto-C also predominantly recognised the smaller p85 Celsr1 species in its more native form whereas cyto-N preferred to ‘pull-down’ the p400 species from the same cell lysate (Fig. 4M). Although we are unable to rule out the possibility that cyto-C is a less sensitive antibody for Celsr1 than cyto-N, altogether the immunocytochemistry (Supplementary Fig. 2C–F), immunohistochemistry (Fig. 4B–J) and Western blot data (Fig. 4K–M) strongly support a hypothesis based on Celsr1 protein variants. We propose an underlying mechanism of differential post-translational modification (Fig. 4N).

### A broad directionality exists in the alignment of core protein asymmetry in skin

**Figure 5:**
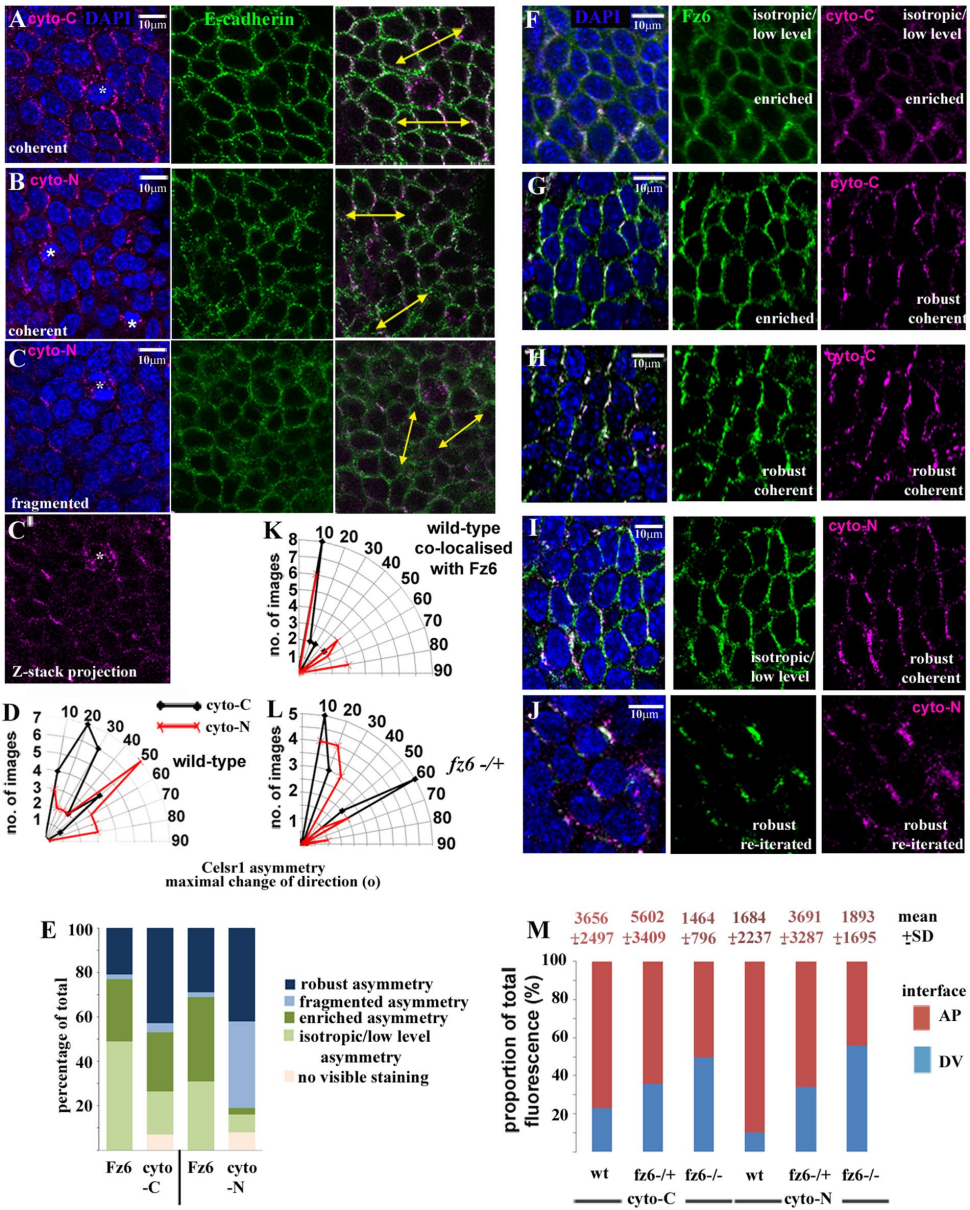
Variation in Celsr1 and Fz6 distribution across the epidermis (**A**–**C’**,**F**–**J**) Asymmetry of core protein distribution along particular body axes is visualised as a zig-zag pattern of antibody staining across the plane of the tissue. Immunostaining of frozen ‘en-face’ sections from E16 C57BL6 wild-type embryos. Anterior is to the left in all images, dorsal is approximately towards the top. Yellow arrows in right hand panels (**A**–**C**) mark the flow of planar polarity across the epidermal progenitor monolayer as visualised by Celsr1 asymmetry. Asterisks in left hand panels label mitotic cells. (**C**,**C’**) representative of 7/14 instances of fragmented Celsr1 asymmetry scored in comparative experiment of cyto-C and cyto-N staining of consecutive skin sections from the same wild-type embryos. (**F**) Fz6 was isotropic in 40% of images (mean of n = 35). (**D**,**K**,**L**) radar plots of the maximal change of direction of Celsr1-cyto-C and Celsr1-cyto-N planar polarisation in wild-type alone (**D**; n = 22 images for each antibody) or when co-localised with Fz6 (K; 14 images for each antibody) and *fz6*+/− (L; n = 15 images for each antibody) with respect to the A-P axis in different confocal images. In (**L**) for 6/15 images we found no visible evidence of cyto-N asymmetry. (**E**) histogram of the relative proportions of different patterns of Fz6 and cyto-C/cyto-N asymmetry when co-localised, n = 25 images. (**M**) proportion of total fluorescence at individual A-P oriented cell interfaces across an epidermal field (30 interfaces from 3 images taken from 3 wild-type and 3 *fz6*−/+ and 3 *fz6*−/− embryos) are shown in red above each bar. Total fluorescence at cell interfaces was normalised against total fluorescence in the centre of the nucleus for each cell analysed. Mean and SD of total fluorescence for each condition is shown in red above each column.

**Figure 6:**
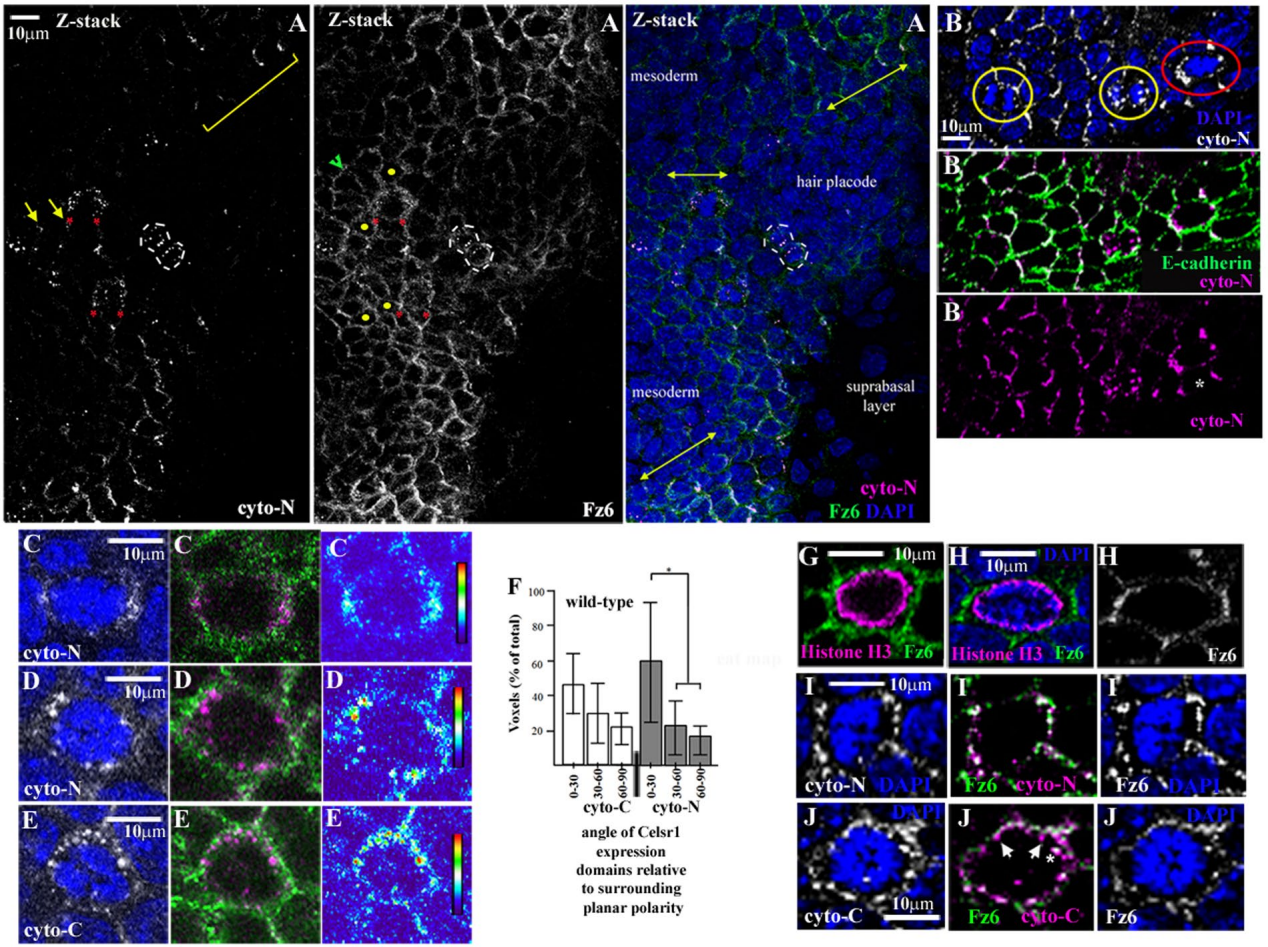
Fz6 and Celsr1 asymmetry is retained during epidermal progenitor mitosis (**A**–**E**,**G**–**M**). Immunostaining analysis, representative images of ‘en-face’ frozen sections. Anterior is the left, dorsal is roughly to the top. Vertical bars in heat maps (**C**–**E**) represent the scale of fluorescence intensity (AU), red is high and blue is low. Representative images are shown. We found cell surface cyto-N asymmetry in early stage mitoses independent of whether surrounding asymmetry in neighbouring interphase cells was coherent or fragmented. (**A**–**E**) wild-type C57BL6 skins n > 3 unless otherwise stated (**A**) ‘en-face’ view of an epidermal field. Yellow double headed arrows (Z-stack reconstruction; right hand panel) highlight the prevailing local axis of planar polarity. Red asterisks label clear cyto-N asymmetry in two early stage mitoses. Yellow dots label neighbouring interphase cells whose long axis is aligned with the axis of local planar polarity. Green arrowhead labels neighbouring interphase cells whose long axes do not align with the local axis of planar polarity. Yellow bracket in left hand panel highlights representative example of cyto-N fragmentation in background of Fz6 isotropy. A mitotic cell in cytokinesis is outlined by dashed white lines. Note the accumulation of both Celsr1 and Fz6 intracellular puncta at the cytokinetic furrow suggesting the polarised reappearance of core protein cell surface asymmetry but along an axis of planar polarity than is almost perpendicular to the prevailing local planar polarity axis. (**B**) 3 basal cell divisions aligned along the A-P axis at different mitotic stages are highlighted by coloured circles. Early phase cell division in red circle exhibits cell surface asymmetry of cyto-N staining which aligns with local axis of planar polarity in neighbouring interphase cells. (**C**) n = 6/16 mitoses (**D**) n = 10/16 mitoses (**E**) n = 20/20 mitoses (**G**) n = 90/125 mitoses (**I**) n = 6/6 mitoses (**J**) asterisk labels intracellular puncta co-expressing Fz6 and Celsr1^cyto-C^ proteins, white arrows label intracellular puncta expressing Celsr1^cyto-C^ proteins but not Fz6. (**F**) histogram showing binned angles of Celsr1 expression domains in early stage mitoses, asterisk indicates two-tailed t-test, (p < 0.01), n > 20 mitoses in each case.

A central tenet of planar polarity is the re-iteration of cellular asymmetry across the plane of a tissue^27^, which can be visualised by a molecular asymmetry of core proteins at opposing cell interfaces within the tissue plane^13, 16, 28^. To investigate whether both cyto-C and cyto-N Celsr1 antibodies exhibit a planar polarised pattern of staining we performed immunohisto-chemistry on consecutive *en*-*face* sections taken across the skin of E16 C57BL6 wild-type embryos and compared them with those of Fz6. Determination of axial orientation is imprecise in frozen sections, thus we used a nomenclature of anterior-posterior (A-P) to denote a head-to-tail direction whereas dorso-ventral (D-V) marked roughly back to belly. Robust asymmetric enrichment of cyto-C and cyto-N to opposing A-P directed cell interfaces but not D-V interfaces was observed (Fig. 5A,B). Fz6 was more often isotropic however irrespective of the Celsr1 pattern (Fig. 5E) suggesting interesting differences in the individual responses of Fz6 and Celsr1 to underlying planar polarity cues. We also found patches of regional enrichment of Fz6 and cyto-C staining (Fig. 5F–J) and a fragmentation of cyto-N staining over a range of a few cell diameters where neighbouring cells exhibited little or no visible evidence of cyto-N staining (Fig. 5C,C’). The latter also correlated with asymmetric Fz6 enrichment (yellow bracket, Fig. 6A). Most striking however were the broad changes in direction exhibited by both Fz6 and cyto-N asymmetry, often approaching 90° i.e. towards D-V (Fig. 5D). In contrast, cyto-C exhibited a narrower directional range (Fig. 5D) as previously described^13, 29^. Fz6 and Celsr1 asymmetry is further quantified in Supplementary Fig. S3A–C. Taken together our analyses expose an unexpected complexity in the distribution patterns of core proteins across the mammalian skin and a broader directionality of both Celsr1 and Fz6 asymmetry than previously recognised. Importantly, the data further argue for the co-existence of variant core protein systems within the mammalian skin basal epithelium.

### Celsr1 and Fz6 retain cell surface asymmetry during mitosis

We next addressed the staining patterns of cyto-C and cyto-N during cell division. We used the gradual reduction in phospho-histone-H3 (PH3) nuclear staining during mitosis to identify different mitotic stages in frozen *en*-*face* sections (Supplementary Fig. S3L–O). Strikingly, cyto-N staining was clearly asymmetric in some early stage mitoses where it aligned with the axis of local planar polarity (red asterisks, Fig. 6A; division in red circle; Fig. 6B) and overlapped with cell surface E-cadherin (division in red circle; Fig. 6B). When we compared cyto-C and cyto-N staining in consecutive *en*-*face* frozen sections taken from the same wild-type embryos we found intense cyto-C staining within intracellular puncta at each mitotic stage (heat map of representative Z-stack image, Fig. 6E; Supplementary Fig. S4) which exhibited A-P bias in late stage (Supplementary Fig. S4, n = 28) but not early stage mitoses (Fig. 6F, n = 25) consistent with previous reports for Celsr1 (Devenport *et al*.^21^). Similarly, cyto-N intensely stained intracellular puncta following chromatin segregation in all mitoses examined (n = 25/25; Supplementary Fig. S4) and in many early stage mitoses (n = 31/40; heat map of representative Z-stack image, Fig. 6D). Again however, in some early stage mitoses cyto-N staining was clearly asymmetric (11/40, 28%) and in n = 4/40 the longest axis of the cell consistently aligned with the axis of local planar polarity (Fig. 6C). That only some mitoses exhibit cyto-N asymmetry can be explained by the different modes of cell division operating in the E16 basal progenitor monolayer: horizontal symmetric divisions are reported to comprise no more than one-third of total cell divisions compared to vertical asymmetric divisions^6^. We subsequently used multiple co-localisation strategies^30^ to illustrate a cell surface distribution of asymmetric cyto-N staining in early stage mitoses (Supplementary Fig. S3S,T). Moreover, we found that asymmetry of cyto-N staining co-localised with the cell surface marker CD44 (Supplementary Fig. S3P,Q). Altogether our findings are consistent with retention of Celsr1 protein asymmetry at cell interfaces prior to mitotic spindle formation in some epidermal mitoses.

Our analysis of core protein asymmetry during interphase was consistent with a novel Fz6-Celsr1 partnership (Fig. 5). To investigate Fz6 patterns during mitosis we undertook a blind analysis of *en*-*face* frozen sections co-stained with PH3 and Fz6. We imaged PH3-positive early stage mitoses and examined Fz6 staining therein. We most often found isotropic Fz6 distribution during early stage mitosis (Fig. 6G), consistent with its interphase pattern (Fig. 5E) but notably, 10/125 mitoses exhibited a planar polarised Fz6 asymmetry in mitoses where the long axis of the cell aligned with the axis of local planar polarity (Fig. 6H). When co-stained, cell surface asymmetric Fz6 and cyto-N staining were co-incident (Fig. 6I). Conversely, cyto-C staining frequently segregated away from cell surface Fz6 within intracellular puncta (arrows Fig. 6J). Thus both Celsr1 and Fz6 exhibit cell surface asymmetry in some early stage mitoses.

**Figure 7:**
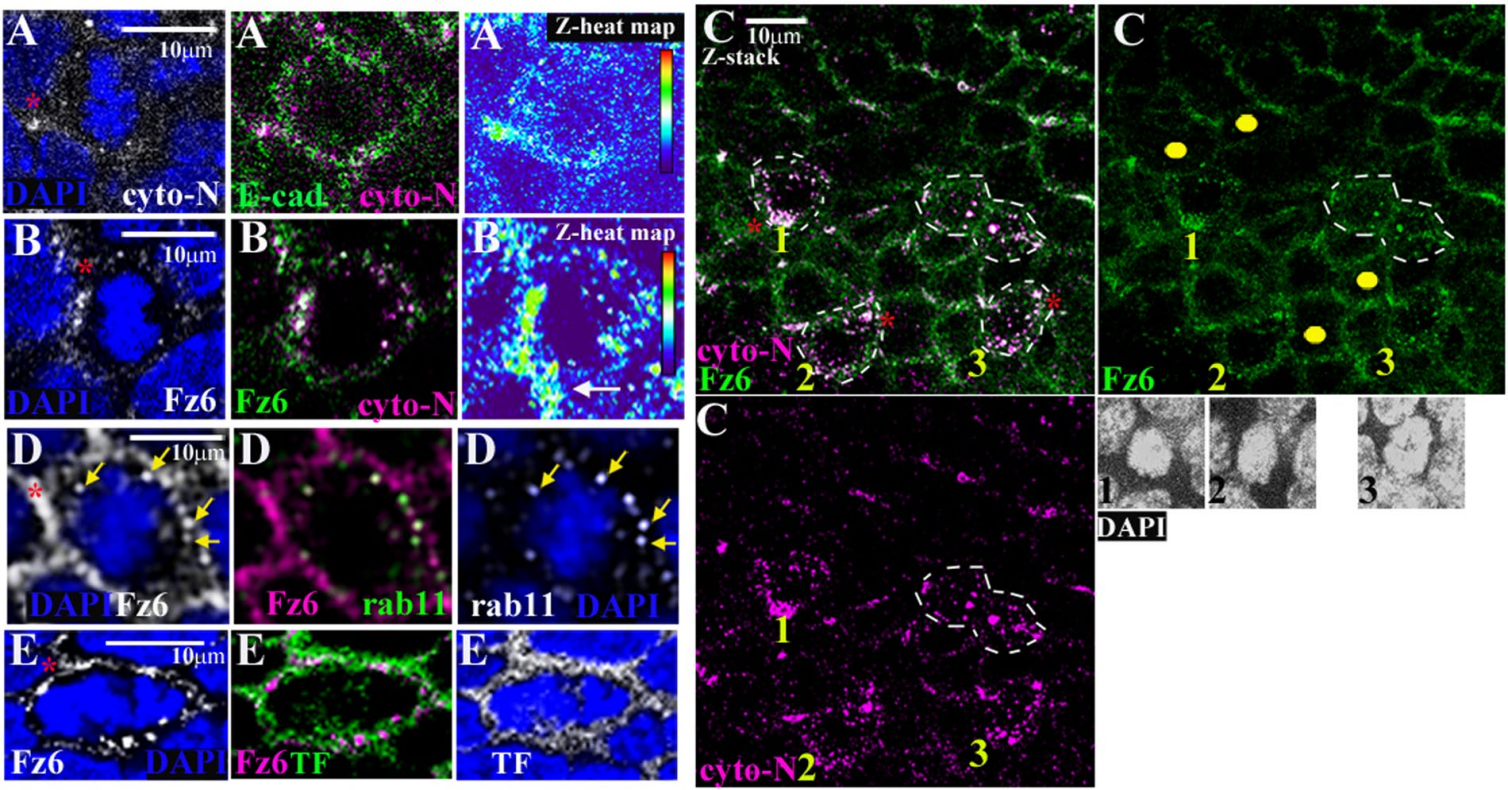
Fz6 and Celsr1 exhibit uni-polar asymmetry as the mitotic spindle forms (**A**–**E**) immunostained ‘en-face’ frozen sections. Mitoses were oriented along the surrounding axis of planar polarity, dorsal is roughly to the top, anterior is left. Z-heat maps show Z-stack reconstructions of Celsr1 distribution during early mitosis (**A**) n = 2, asterisk labels uni-polar focal enrichment of Celsr1 (**B**) n > 3, asterisk labels uni-polar focal enrichment of Fz6 and Celsr1 at interface with neighbouring interphase cell, arrow labels Celsr1 enrichment in interphase neighbour (**C**) representative image, n = 3. Three early stage mitoses, labelled 1,2,3 together with a mitosis in cytokinesis (white dashed lines) whose plane of cell division is perpendicular to the axis of local planar polarity marked by cyto-N and Fz6. Yellow dots label neighbouring interphase cells with a long axis aligned with the axis of local planar polarity. Z-stack reconstructions of the nuclear mass for each early stage mitosis are shown underneath the right hand panel. (**D**) arrows label intracellular puncta co-expressing Fz6/rab11, n = 3. (**E**) TF is transferrin, n = 4, asterisk labels uni-polar Fz6 cell surface enrichment.

In addition to bi-polar (enrichment to opposing cell interfaces) Celsr1 and Fz6 cell surface asymmetry during early stage mitosis (Fig. 6B,C,H) we additionally uncovered evidence for a unipolar pattern. This manifested as either little or no apparent antibody staining, even in Z-stack images (n = 2/40 cyto-N stained mitoses), or an accumulation of Celsr1 and Fz6-containing intracellular puncta (n = 1/40 for cyto-N-stained mitoses; 7/125 for PH3/Fz6 double-labelled mitoses), on the opposite side of the cell to core-protein cell surface enrichment (Fig. 7A,B respectively). This ‘uni-polar’ asymmetry aligned with the local planar polarity axis in neighbouring interphase cells, both coherent and fragmented variations (Fig. 7A,B) and, strikingly, distinguished both the head and the tail of the embryo (n > 3, representative examples shown in Fig. 7C). Where found, Fz6-containing intracellular puncta located opposite to Fz6-enriched cell interfaces co-labelled with rab11 (arrows, Fig. 7D) revealing them as recycling endosomes, which also sequester Celsr1 proteins at the onset of mitosis^21^. Co-staining with transferrin revealed that some Fz6 might also traffic into the degradation pathway (Fig. 7E). Taken together our data reveal the existence of different modes of core protein cell surface asymmetry during the early stages of skin basal cell mitosis. Importantly, Celsr1/Fz6 distribution is polarised at the mitotic cell surface at the right time for mitotic spindle capture.

### Evidence that core protein cell surface asymmetry connects cell division plane with neighbouring interphase cell long axis geometry

**Figure 8:**
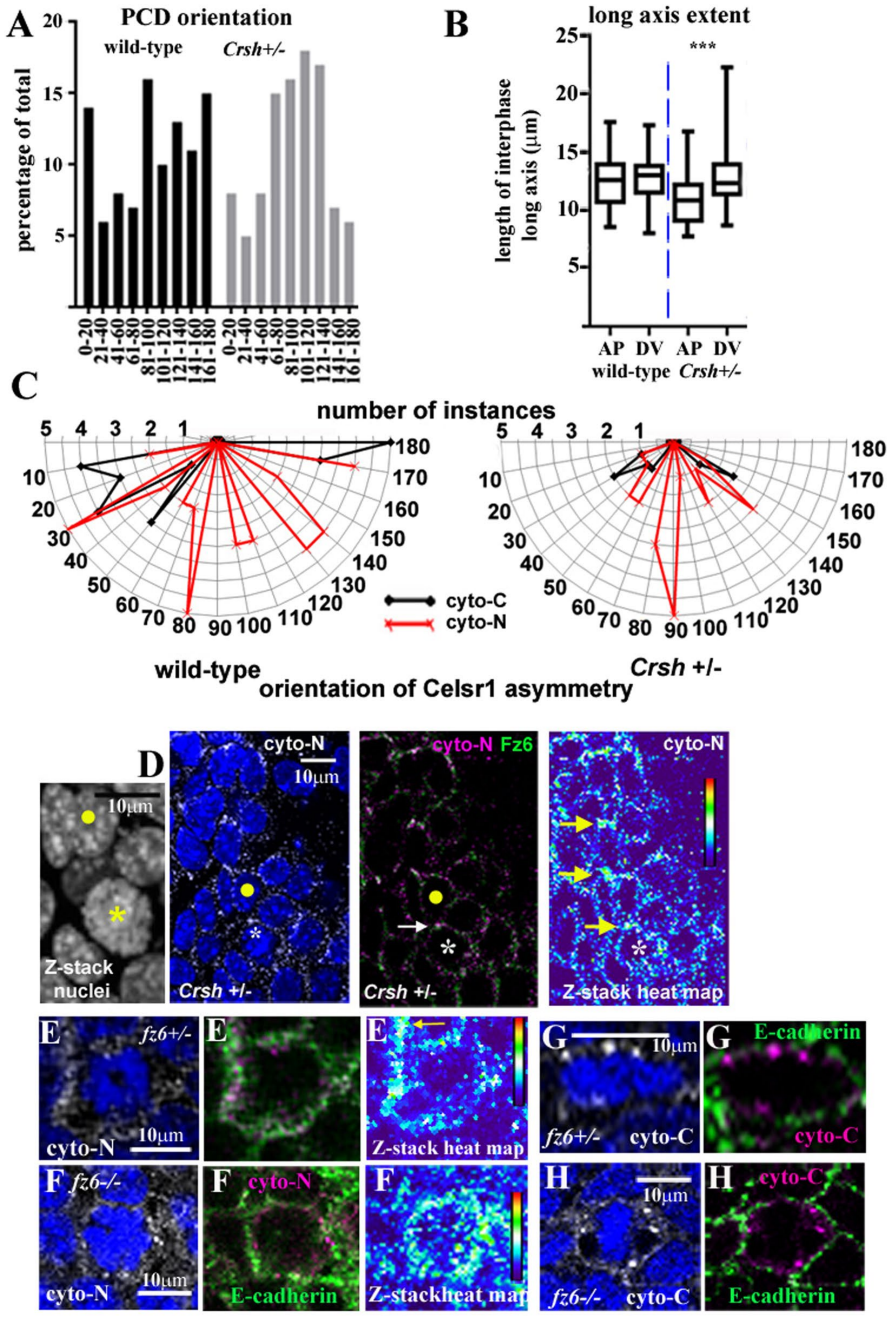
Evidence that core protein asymmetry aligns cell division plane with the long axis extent of interphase neighbours. All representative images are shown with anterior to the left and dorsal to the top. (**A**,**B**) histograms and whisker box plots of PCD orientation and long axis extent (as shown previously in Fig. 3). (**C**) radar plots of the range of axial bias in Celsr1 asymmetry in wild-type or *Crsh* heterozygotes (n = 14 images for each antibody taken from 3 embryos for each condition from 2 different litters). (**D**) *Crsh* heterozygote skins, n > 3, asterisk labels mitotic cell, yellow dot labels interphase neighbour. Yellow arrows mark re-iteration of cyto-N asymmetry along the DV axis (**E**,**F**) n > 3 mitoses for each antibody, arrow in (**E**) labels planar polarised enrichment of cyto-N in neighbouring interphase cell. (**G**,**H**) n > 3 mitoses for each antibody. **‘**En-face’ frozen sections were immunostained and analysed blind.

The data above argues that cell surface asymmetry of core proteins prior to mitotic spindle formation directly links cell division plane with neighbouring interphase cell shape. We would expect therefore that *Crsh* heterozygotes, in which DV oriented interphase long axis extent strongly correlates with DV PCD orientation, would exhibit alignment of core protein cell surface asymmetry along the same skin axis (Fig. 3). Conversely in *Crsh* and *fz6* KO homozygotes, where we find little correlation between long axis geometry and PCD orientation, we would expect loss of core-protein asymmetry (Figs 2 and 3). This is indeed the case (Fig. 8, Supplementary Fig. S3H–K). In *Crsh* heterozygotes, Celsr1 was very often isotropic during interphase (data not shown). When asymmetry was observed, for both cyto-C and cyto-N, it was poorly re-iterated across the skin epithelium but importantly, cyto-N exhibited a strong DV bias both during interphase and mitosis (Fig. 8C,D) compared to its broader directionality in wild-type (Fig. 8C). Moreover, Fz6 enrichment mirrored the cyto-N fragmented pattern (Fig. 8D and data not shown). Celsr1 was also isotropic during progenitor cell mitosis in *Crsh* homozygotes (Supplementary Fig. S3J) consistent with a previous study of interphase cells in this mutant^13^.

We also found that robust asymmetry of cyto-C and cyto-N staining during interphase progressively disappeared in *fz6* KO embryos (Fig. 5M; Supplementary Fig. S3E–G) consistent with the inter-dependence of core protein asymmetry previously reported in Drosophila^31^ and during skin development^13^. Importantly, in *fz6*+/− skins, the novel fragmented asymmetry of cyto-N staining and its broad directional range mostly disappeared (Supplementary Fig. S3E; Fig. 5L). Indeed, cyto-N asymmetry now appeared more similar to cyto-C. Thus the novel variant patterns of Celsr1 protein asymmetry across the epidermis depend upon Fz6. In *fz6*+/− mitoses cell surface cyto-N asymmetry aligned with the local axis of planar polarity in neighbouring interphase cells (arrow, Fig. 8E). Celsr1 symmetry during mitosis was not robust however, as cyto-N staining also clearly labelled D-V cell surfaces (Fig. 8E) which would explain the lack of axial bias in PCD orientation in this mutant (Fig. 2B,B’). In *fz6*−/− skins, cyto-N asymmetry was lost in mitotic progenitors: antibody staining localised around the entire cell surface as well as within intracellular puncta (Fig. 8F). Thus cell surface asymmetry of cyto-N staining during early mitosis is also Fz6-dependent.

Altogether these data support a model whereby an asymmetric cell surface cue aligns the cleavage plane of a mitotic skin progenitor cell with the long axis extent of its interphase neighbour(s) so that both align along the same axis of local planar polarity.

Finally, it was intriguing that despite the limited re-iterative and robust asymmetry of Celsr1 in *Crsh* heterozygotes, long axis geometry remained linked to PCD orientation, raising the question of whether the long axis geometry of one interphase neighbour, when linked to a mitotic basal cell via asymmetry of Celsr1/Fz cell surface enrichment at a single common interface, was sufficient to orient a mitotic spindle. To address this we analysed mitotic cells exhibiting either bipolar or unipolar Fz6/Celsr1 asymmetry where the entire long axis of each neighbouring interphase cell was fully visible in confocal Z-stack images (n = 10 and 6 respectively). We asked what proportion of interphase neighbours surrounding the mitotic cell exhibited core protein asymmetry across their longest axis. On average it was just under half (47%+/− 23%; n = 3–7 neighbours/mitotic cell, average of 5.4) with a minimum of one interphase neighbour in n = 2 mitoses. These data support the hypothesis that the long axis of an interphase neighbour orients the cell division plane of a mitotic cell when both cells are connected by an asymmetric core protein-dependent cell surface cue.

## Discussion

**Figure 9:**
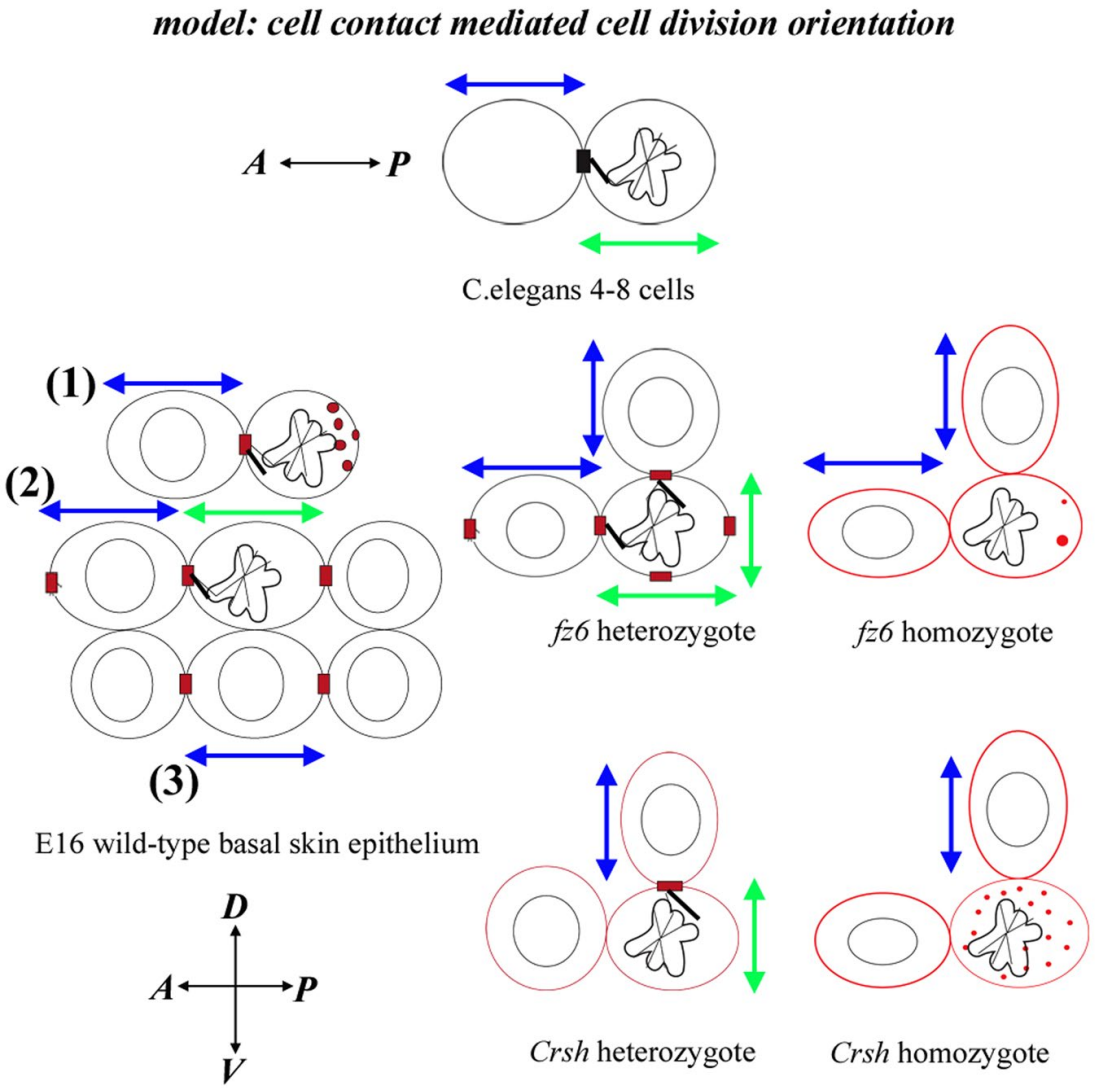
Model of core protein dependent cell-contact mediated orientation of cell division in the mammalian skin. Taken together our data suggest that in wild-type the asymmetric cell surface cue which aligns cell division plane with neighbouring interphase long axis extent can be uni-polar (enriched at the interface between mitotic cell and the interphase neighbour) as shown in division 1 or bi-polar as shown in division 2,3. The asymmetric cue can be located ‘head-on’ with the mitotic cell as shown in division 1 and 2 or alongside the mitotic cell as shown in division 3. A: anterior, P: posterior, D; dorsal V; ventral. Blue arrows denote interphase long axis orientation, green arrows denote PCD orientation. In *C*. *elegans* cartoon, black box labels Wnt/Frizzled cell surface cue. Red boxes label Celsr1/Fz6 cell surface asymmetry. Black lines denote putative link between the cell contact cue and the mitotic spindle. Red dots denote Celsr1/Fz6-expressing intracellular vesicles. In core protein mutants Celsr1 planar polarised asymmetry during interphase diminishes as marked by red cell outlines.

The interphase cell long axis is linked to cell division orientation in a number of different cell and tissue contexts^2, 5, 32^. Intriguingly, planar polarity proteins have previously been reported to resist the ‘long-axis’ rule and instead align cell division planes along perpendicular axes to cell long axis elongation in neighbouring interphase cells^9^. Studies of the insect wing also reveal that planar polarity-dependent control over cell division orientation can be achieved by actively generating cell shape anisotropies in the mitotic cells themselves^12^. In the mammalian skin we propose a new variation, which relies on the long axis geometry of interphase neighbours. We provide compelling evidence from *Celsr1* and *fz6* mouse mutants that core protein asymmetry connects a process of cell contact-mediated orientation of skin planar cell division (Fig. 9). Altogether these data suggest a model where retention of a planar polarised, cell-contact mediated cortical cue aligns skin mitotic spindles with the long axis of an interphase neighbour along the same axis of local planar polarity. In cases of bi-polar core protein asymmetry however we cannot rule out additional cell autonomous effects on cell shape. Notably, cell-contact with interphase neighbours specifically orients horizontal divisions in the early 4-cell *C*. *elegans* embryo (reviewed in ref. 33 and Fig. 9). Elegant cell isolation experiments demonstrated that Wnt/Src-dependent signalling transmits a polarised cortical signal from an ‘inducing’ cell to a ‘responding’ cell to align the subsequent cell division of the responding cell along the A-P axis^34, 35^. In this context, polarised signals are transduced downstream by the Frizzled receptor MOM-5^36^. Notably, A-P oriented cell divisions from the 8-cell stage also depend upon the Latrophilin orthologue *lat*-1, acting in parallel with Wnt/Fz signalling^37^. Latrophilin is a member of the Adhesion-GPCR (aGPCR) superfamily and bears structural similarities in its GPCR-domain to Celsr1^38^. The nematode Celsr1 orthologue, *fmi*-*1*, does not seem to play a role in planar cell polarity however raising the hypothesis that *lat*-*1* may perform the same role as Celsr1 in nematode planar polarity including the Fz-dependent orientation of horizontal cell division. Celsr1, also an atypical cadherin, has the potential to form homophilic protein complexes with itself on opposing cell interfaces as well as signal into the cell. It is a strong candidate therefore for ‘bridging’ between mitotic and neighbouring interphase cell(s). Whether the MuD/Dishevelled/Dynein pathway, previously reported to be downstream of Fz in cell division orientation in other tissue contexts^11^, has any relevance here remains to be determined. Notable here is that Fmi/Stan or Celsr protein function in Fz-dependent cell division orientation has not been previously described^11, 39^. Intriguingly, lat-1 is reported to orient the plane of *C*. *elegans* cell division via signalling from its GPCR-like domain^40^. Thus, Celsr1 might also utilise GPCR-specific signalling pathways to orient skin cell divisions.

Our data raise a further hypothesis that the axial bias in skin spreading is reinforced during embryonic growth via alignment of planar cell shape and horizontal cell division downstream of the core planar polarity pathway. Although on a different scale, the local amplification of small biases in global planar polarity across a tissue is central to core protein function^41^. The implication of our work is that in the skin, planar cell shape is disconnected from skin planar cell division in the same cell. This mechanism may derive from the greater complexity of skin core protein distribution described here compared to, for example, Ft/Ds/Dachs distribution in the insect wing^12^. Notably, the mouse mutants we examined suggest that core proteins also impact on axial bias in interphase long axis geometry (Figs 2 and 3). Their precise roles in this important process are however currently unclear.

Nevertheless, skin planar polarity recapitulates many of the cellular and molecular features of core planar polarity processes in the insect wing^13, 21, 42^. It is highly relevant here that Prickle protein isoforms drive variation in core protein signalling in the latter to concomitantly align wing hairs and wing cuticle ridges along perpendicular wing axes^43^. Intriguingly, we found that in E16 wild-type skins from two different mouse strains, HF and PCDs effectively align their orientation along roughly orthoganol A-P oriented vectors (Supplementary Fig. S1; Figs 2 and 3), which notably fall within our designated AD and AV quadrants (Fig. 1). Notably, cyto-C staining is consistent with reported roles for Celsr1 in the local re-iteration of uni-directional planar polarity linked to HF alignment^13, 21^. The directional pattern of cyto-N asymmetry is more complex however, spans multiple body axes and is linked here to both planar cell shape and PCD orientation. Moreover, directional specificity for each of these components is lost in *fz6*+/− skins whereas HF alignment remains similar to wild-type (Supplementary Fig. S1). The latter might be explained by the directional pattern of cyto-N asymmetry in *fz6*+/− skins, which more closely mimics that of cyto-C (Fig. 3L). *Crsh* heterozygote HF phenotypes however (Supplementary Fig. S1) argue that HF orientation is also complex and potentially linked to global patterns of Celsr1/Fz6 planar polarity. Importantly these observations provide further support for the hypothesis that variant Celsr1 proteins co-exist across the mammalian skin epithelium, raising the intriguing question of whether they could elicit unique responses to the same or possibly different skin planar polarity signals.

The intracellular accumulation of core proteins during mitosis is, as far as we are aware, unique to the mammalian skin. We show here that the timing of this accumulation is Fz6-dependent and specific for different Celsr1 antibodies. If our antibodies do distinguish Celsr1 protein variants, the differential accumulation of cyto-C-positive intracellular puncta during early stage mitosis may reflect the necessity for their spatial separation during skin basal cell mitosis to ensure a robust, short-range polarising signal for PCD orientation as well as maintenance of longer-range planar polarity across the epidermis as previously proposed^21^. Indeed the Celsr1 antibody used in the Devenport study is highly similar in staining pattern to cyto-C, which further argues for Celsr1 protein variation underpinning cyto-C and cyto-N differences. It is noteworthy here that the C-terminal tail of the partial Celsr1 tail protein we used to affinity purify cyto-N includes protein motifs linked to the phosphorylation-dependent intracellular accumulation of Celsr1 at the onset of mitosis^44^. In addition to the differential staining patterns of cyto-C and cyto-N, we were equally intrigued by the apparently fragmented patterns of Celsr1 reminiscent of Flamingo distribution in the proliferating epithelium of the insect larval wing^45^. It is clear therefore that much is still to be uncovered with respect to core protein function in skin planar polarity. Moreover the exact nature of the putative Celsr1 protein variants and their potential impact on the epithelia of other mammalian tissues and organs including the nervous system and lung remains an additional but exciting challenge for the future.

## Methods

### Ethics statement

Animal husbandry and embryo dissections were approved by the UK Home Office and performed according to UK Home Office laws and guidelines.

### Mice and embryo dissections

The *Celsr1* mouse mutant *Crsh* (BALB/c) and the *fz6* KO mouse (129/C57BL6) have been described previously^18, 46^. *Fz6*−/− males were backcrossed to C57/BL6 wild-type females (Charles River). Heterozygote KO males and females were mated to generate wild-type, heterozygote and homozygote embryos for immunostaining analysis. All adult mice were genotyped by PCR. Timed matings were performed and 9am of the first day of plugging was taken as E0.5. Immunostaining for Celsr1 protein isoforms was performed on CD1 wild-type strain unless otherwise specified. Pregnant females were culled using a Schedule 1 procedure. All embryos were dissected away from the uterus and decapitated immediately prior to fixation and immunostaining analysis.

### Generation of Celsr1-protein isoform specific polyclonal antibodies

The full length Celsr1 cytoplasmic tail (FLcyto) was cloned into a Qia-express vector and expressed in bacteria as a His-tagged protein of around 42 kDa before purifying the expressed protein using TALON polyhistidine-Tag purification resin (Clontech) as recommended by the manufacturer. Purified protein was used directly to generate rabbit polyclonal antibodies (Eurogentech). Rabbit sera was first affinity purified against Ncyto protein as described^20, 25^. Sera was collected as it vacated the column and was loaded directly onto a second column containing FLcyto protein coupled to Affi-gel-15 agarose. Both columns were washed and the specific antibodies eluted as described previously^20, 25^. Antibody affinity purified by Ncyto protein was labelled cyto-N. The second affinity purified antibody fraction was labelled cyto-C. Ncyto and FLcyto proteins were used to show specificity of each antibody fraction by western blotting. Briefly, both proteins were independently run in a single large well and the separated proteins transferred to PVDF membrane (BioRad). Strips were cut from each blot and incubated with pre-immune sera alone, immunised whole rabbit sera alone, and titrated amounts of affinity purified cyto-N or affinity purified cyto-C antibodies. Whereas cyto-N recognised both Ncyto and FLcyto proteins, cyto-C only recognised FLcyto, even at the lowest dilutions, demonstrating its specificity for the most c-terminal part of the FL Celsr1 cytoplasmic tail (Fig. S2A). Specificity for Celsr1 in mouse tissues was tested by pre-absorption of both affinity purified Celsr1 antibodies with FLcyto for 30 min at room temperature. Following a brief centrifugation step, supernatant was incubated with frozen sections of mouse spinal cord which had previously undergone fixation, blocking and several washes. Whilst staining for Celsr1 protein was apparent following incubation with untreated cyto-N and cyto-C, no signal was detected with the pre-absorbed sample (Fig. S2B). Antibody specificity was further confirmed through loss of planar polarised asymmetry of antibody staining in the epidermis of the *Celsr1* mouse mutant, *Crash* (data not shown).

### Western blots

Western analysis was as previously described^20^.

### Frozen sections

Decapitated wild-type and mutant mouse embryos were either fixed overnight in 4% formaldehyde/PBS and incubated in 30% sucrose/PBS until the embryo body sank prior to embedding in OCT (VWR) or embedded immediately. Embryos were embedded with their backs down and their limbs facing upwards. Longitudinal sections were taken using a Micron Cryostat. Multiple frozen sections were taken from beyond the shoulder in a dorsal-ward direction.

### Wholemount flank skin preparations

Decapitated wild-type and mutant mouse embryos were fixed overnight in 4% formaldehyde/PBS. Following fixation the embryo trunk was isolated away from the rest of the embryo body and flank skin was dissected by cutting along the spinal cord on both sides. Flank skin, including ventral regions surrounding the umbilical cord, was peeled away as one piece of tissue to generate an open book preparation.

### Antibodies and immunohistochemistry

Cytokeratin 10 antibody (1:1000, Thermo-scientific RKSE60), cytokeratin 1 antibody (1:500; Covance AF109), cytokeratin 14 antibody (1:1000; Covance), cytokeratin 15 antibody (1:500; Millipore), fibronectin (1:800, Sigma; F3648), E-cadherin (1:4000, DECMA-1; Sigma), vinculin (1:1000, Sigma), Ki67 (1:100, Abcam ab16667), Histone H3 (1:300, Millipore), Frizzled6 (1:100, R&D systems), Myosin II heavy chain (1;500: Sigma), Myosin light chain (1:50: Cell signalling), anti-Myosin pS19/pS20 (1:500, Rockland), active Caspase-3 (1:500, R&D Systems). *Frozen sections*: Immunofluorescence analyses for Celsr1 on frozen sections were as described (Formstone *et al*.^20^). Immunofluorescence analyses for antibodies other than Celsr1 involved fixation of embryos overnight and subsequently incubation in 30% sucrose/PBS until the embryos sank prior to embedding in OCT (VWR). Immunostaining was then performed as for Celsr1. *Wholemount immunostaining* was essentially as described^13^. Dissected skins were first fixed for 1 hour in 4% formaldehyde/PBS and washed with PBS. Sections and whole tissue were mounted in Mowiol (0.25 g/ml mowiol, 33% v/v glycerol, 0.005% v/v DABCO) and imaged on a confocal microscope (Nikon confocal, A1R). Images were analysed using *Image J* or Volocity software (Perkin Elmer).

### Quantitative analyses on immunostained frozen sections

All analyses on the *fz6* KO litters were performed blind. Genotyping of tail tissue by PCR was performed following analysis to verify the genotype of each embryo. Immunohistochemistry was performed on 30 µm thick longitudinal frozen sections. *Changes in direction of planar polarity* across an epidermal field relative to the A-P axis were measured using the ruler tool in Adobe Photoshop. *Quantification of angle of Celsr1*-*expression domains in mitotic cells* Individual mitoses were cropped in Volocity software and orientated so that anterior-posterior axis was left to right in the image. In the measurement window, Celsr1 expression domains and the nucleus were made objects and a snapshot was taken of each Celsr1 object. In Adobe Photoshop the centroid of the condensed nuclear chromatin was used as the pivot to measure the angle from the centroid of each Celsr1 object relative to the left right axis of the image (directional flow of planar polarity). For late stage divisions the centre of the closest condensation of chromatin was used (see Fig. S3A). Angles were plotted as polar plots (late stage divisions) or as a histogram (early stage divisions). Statistical analysis was two-paired t-test. *Van Steensel’s cross*-*correlation approach for co*-*localisation of cell surface receptors*. Co-localisation studies are often enigmatic but Van Steensel’s approach^47^ was preferred because it was specifically designed to co-localise punctate clusters of cell surface receptors using a cross-correlation analysis which distinguishes between co-localisation and exclusion and takes into account unrelated signals i.e. background noise which would be expected to be a problem when using immunostained tissue^30^. We also cross-referenced this value with that of Costes’ which was also calculated in JACoP (Image J plug-in ref. 30); and evaluates the correlation coefficients based on stochastic colocalisation events and scored Van Steensel’s value only when it agreed with that of Costes’. *Nikon imaging software intensity plots*. Intensity plots were generated along A-P and D-V interfaces across a continuous field of epidermal progenitor cells (minimum 10 cells). Total levels of fluorescence at individual cell interfaces were calculated in excel (Microsoft). *Heat maps* were generated using the *Image J* plug-in *heat map histogram*.

### Quantitative analyses on immunostained wholemount skins

All analyses on *fz6* KO litters were performed blind. Genotyping of tail tissue by PCR was subsequently performed to verify the genotype of each embryo. *Quantification of the orientation of planar cell divisions*: Anterior, posterior and mid-regions of dorsal flank skin were imaged and then analysed independently. 10–15 sequential images were taken on a Nikon A1R confocal at 60X magnification. Z-stack images were generated using 0.3 µm steps. 3-D reconstructions (Volocity software) of each Z-stack were generated for each image. All telophase divisions and the underlying basal lamina were individually cropped and the resulting images rotated (3D opacity mode) until the segregation of each chromatid pair could be measured relative to the basal lamina (marked by staining for fibronectin). One snapshot was taken from different sides of any given division. Angles of cell division relative to the basal lamina were measured using the *ruler* tool in Adobe Photoshop and the mean angle for each division was calculated. Division orientation in the x-y axis (planar orientation) was subsequently measured for mitoses which were oriented at 28° or less from the basal lamina (Lechler and Fuchs, 2005). Images were again rotated in Volocity and the angle of cell division orientation with respect to the plane of section was measured using the *ruler* tool in Adobe Photoshop. *Mitotic cells* were scored by intensity of nuclear DAPI staining. Planar orientation was then measured by exporting an XY snapshot into Adobe Photoshop. The angle of chromatin segregation relative to the rostro-caudal axis was then measured using the ruler tool. The total sum of particular orientations for each experimental group was then plotted.

## Electronic supplementary material

Supplementary information
