# Supplementary material for: A role for core planar polarity proteins in cell contact-mediated orientation of planar cell division across the mammalian embryonic skin
Source: Sci Rep. 2017 May 12;7:1880. doi: 10.1038/s41598-017-01971-2 (PMC5431842; doi:10.1038/s41598-017-01971-2)
Supplement: Supplementary file 1 — Supplementary information [file 41598_2017_1971_MOESM1_ESM.pdf]

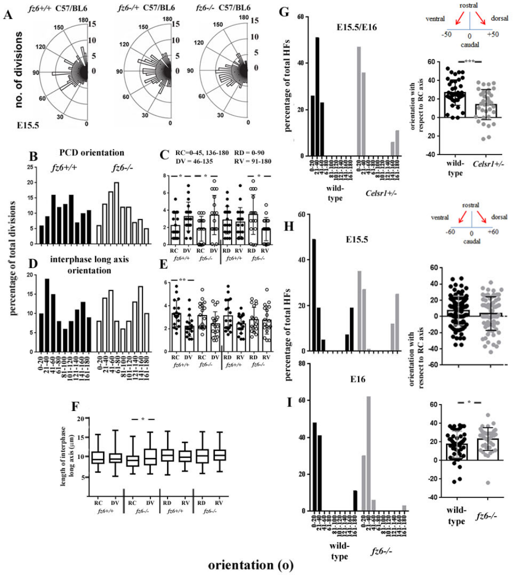

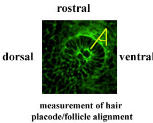

Supplementary Figure 1:

Orientation of PCD and HF down-growth in E15.5-E16 *fz6* and *Celsr1* mouse embryo skins. **(A)** polar plots of PCD orientation in E15.5 *fz6* KO litters. **(B-E)** histograms of PCD orientation (B,C) and interphase long axis orientation (D,E), see legend for Figure 1 for detailed explanation. **(F)** Whisker box plot of interphase long axis extent. RC is rostro-caudal; DV is dorso-ventral: RD is rostro-dorsal, RV is rostro-ventral orientations in *Crsh* (G) and *fz6* KO littermates, again see legend of Figure 1 for detailed explanation. **(G-I)** histograms (left) and scatter plots (right) of hair follicle orientation in *fz6* KO and *Crsh* mutant littermates. Statistical analysis was Student's t-test, * denotes P<0.01, ** denotes P<0.001. *** denotes P<0.0001. Schematic shows the methodology used to measure hair placode and hair follicle orientation with respect to the rostro-caudal (RC) axis of the dorsal flank.

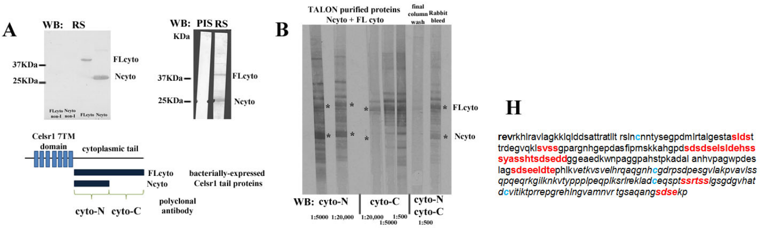

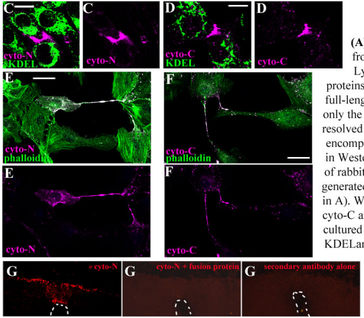

Supplementary Figure 2: validation of Celsr1-specific antibodies

(A) Western blot analysis of bacterially-expressed tail proteins against whole sera from rabbits (RS) immunised with a full-length Celsr1 cytoplasmic tail protein. Lysates from bacterial preparations induced to express different sized Celsr1 tail proteins were run alongside controls which were non-induced preparations (non-I). The full-length Celsr1 tail protein (FLcyto) resolved at around 42KDa. A protein encompassing only the N-terminal portion (Ncyto) of the Celsr1 tail (Formstone et al, 2010; Qu et al, 2010 resolved at 28KDa. Schematic shows relative portions of the Celsr1 cytoplasmic tail encompassed by each protein. **S**era from FLcyto immunised rabbits recognised both proteins in Western blots, pre-immune sera (PIS) did not. **(B)** Affinity purification of the same batch of rabbit sera against Ncyto protein and subsequently against FLcyto (see methods) generated two distinct polyclonal antibodies, cyto-N and cyto-C respectively (see schematic in A). Whereas cyto-N antibody recognised both Ncyto and FLcyto (see schematic in A), cyto-C antibody recognised FLcyto but not Ncyto. **(C-F) i**mmuno-histochemistry of cultured L cells (C,D) and MDCK cells **(E,F)** stably expressing a full-length Celsr1 cDNA. KDELand phalloidin mark golgi and F-actin respectively. Both cyto-Nand cyto-C antibodies recognise a protein which enriches at cell interfaces both in L-cells, which normally exist as isolated cells in culture due to their lack of adhesion components, and in adhesive MDCK cells. **(G)** cyto-N antibody staining was lost from the roof plate of the E13.5 mouse spinal cord following pre-incubation of affinity purified rabbit sera with Ncyto bacterially expressed fusion proteins prior to immunohistochemistry. (**H**) **a**mino acid sequence of Celsr1 cytoplasmic tail. Bold regular text shows region used to affinity purify cyto-N, text in italics denotes region corresponding to cyto-C. blue labels potential palmitoylated cysteines, red labels potential phosphorylation sites.

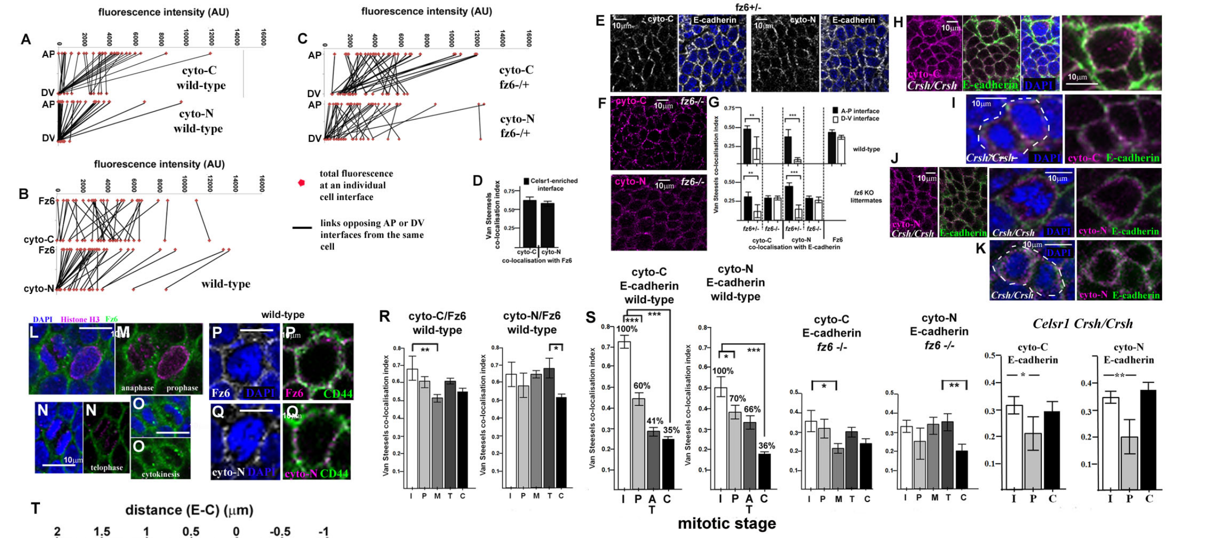

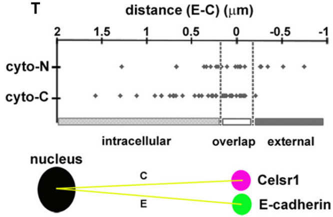

Supplementary Figure 3: quantification of Celsr1 protein variant distribution in the skin epithelium during interphase and mitosis. (A-C) Measurements of fluorescence intensity at individual cell interfaces, AP denotes anterior-posterior, DV denotes dorso-ventral n>25 interfaces from 4 images taken from at least 3 different embryos in each case. Fluorescent intensity at cell interfaces was normalised against fluorescent intensity at the centre of the nucleus. In (A), bottom panel, cyto-N: low or no fluorescence at multiple cell interfaces was consistent with fragmentation of cyto-N staining (B) Staining levels for Fz6 and cyto-C were similar at individual A-P interfaces across a field of cells. whereas many Fz6-expressing A-P oriented interfaces showed no visible cyto-N staining. Notably, where cyto-N staining was not visible, Fz6 staining was mostly isotropic. (C) both cyto-C and cyto-N staining exhibited a mixture of anisotropic enrichment to opposing cell interfaces and isotropic cell surface expression in *fz6* +/- skins. **(D)** co-localisation index of Fz6 with either cyto-C or cyto-N staining, mean and SD are shown. We used Van Steensel's co-localisation approach (Van Steensel et al, 1996; Bolte and Cordelieres, 2006 and methods).

(E,F) cyto-C and cyto-N staining in heterozygote *fz6* KO skins, anterior is to the left, dorsal is to the top. **(G)** histogram showing the co-localisation indices (Van Steensel) of cyto-C and cyto-N respectively with Fz6 in progenitor cells exhibiting Celsr1 asymmetry. Mean and SEM are shown. >15 cells were analysed in each case. Asterisks indicate two-tailed t-test, ** p<0.001, *** p<0.0001. **(H-K)** cyto-C and cyto-N staining in Celsr1 *Crsh* homozygote skin during interphase and mitosis. White lines mark basal cell at cytokinesis. **(L-O)** Phospho-histone H3 labelling of skin basal cells during mitosis as a marker of progression in mitosis. **(P,Q)** Fz6 (P) and cyto-N (Q) staining co-labels the CD44-positive cell surface during ear;y mitosis. Representative images, n>3. **(R,S)** protein co-localisation was quantified using JaCOP, an intensity-correlation-coefficient-based (ICCB) approach available in Image-J (Bolte and Cordelieres, 2006) used previously to quantify co-localisation of Celsr1 with E-cadherin at the plasma membrane of epidermal progenitor cells (Devenport et al, 2011). We used Van Steensel's approach however, not the Manders' coefficient (see Methods for explanation). I; interphase, P; prophase, M; metaphase, A; anaphase, T; telophase, C; cytokinesis. n>10 cells for each stage. Asterisks indicate two-tailed t-test, * denotes p<0.01, ** denotes p<0.001, *** denotes p<0.0001. (S) percentage value above each bar represents proportion of co-localisation compared to interphase (100%). **(T)** we also used an object-based analysis (Bolte and Cordelieres, 2006) to measure the distance between the centroid of the nucleus of each cell in prophase and the centroid of individual neighbouring Celsr1 (C) and E-cadherin (E) domains. Schematic shows method of measurement. Value C is the distance (μm) between the centroid of an individual Celsr1 expression domain and the centroid of the nucleus. Value E is the distance (μm) between the centroid of a nearest neighbour E-cadherin expression domain and the same nucleus. The difference between these measurements (E-C) was calculated and is shown as a scatter plot. Those which fell below the optical resolution of the confocal microscope (estimated as 0.3μm based on a 63X oil immersion Plan Apochromat objective, NA 1.4) were assigned as overlapping. Higher E-C values for cyto-C (mean of 0.38) supported an intracellular distribution whereas lower E-C values for cyto-N (mean of 0.16) argued that these Celsr1 protein variants were not retained within intracellular vesicles during mitosis. Unexpectedly, several cyto-N-expressing domains located beyond their nearest neighbouring E-cadherin domain (i.e. some Celsr1 proteins were further from the nucleus relative to E-cadherin). Possibly, these data reveal cell surface Celsr1 versus recycling E-cadherin/Celsr1 populations at the cortex

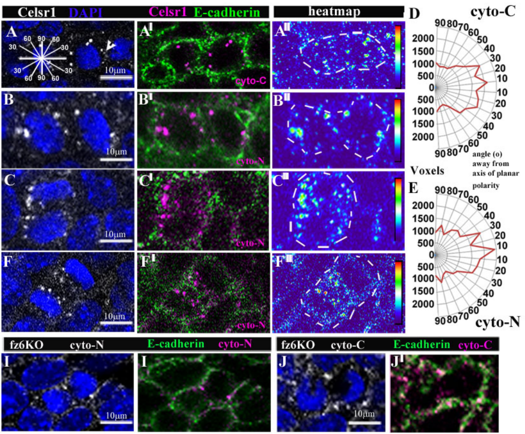

Supplementary Fig.4: All Celsr1 protein variants appear in intracellular vesicles following chromatid segregation. Their intracellular distribution at telophase/cytokinesis is biased along the A-P axis (D,E). They re-appeared at the plasma membrane at cytokinesis as described previously for Celsr1 (Devenport et al, 2011). **(A-F, G-J)** immunostaining of frozen sections, representative images n>3. (A) arrowhead labels cell surface Celsr1. The method used to measure the angle of distribution of Celsr1-fluorescence within intracellular puncta during telophase and cytokinesis with respect to the axis of surrounding planar polarity, as shown in **(D,E)**, is indicated in **(A)**. n> 30 mitoses in each case. (F-F'') representative image, n=6/36 cyto-C and cyto-N-stained divisions. white dashed lines mark the edges of the mitotic cell. Anterior is towards the left, dorsal is roughly towards the top in all images.
